# Effective Partnership in Community-Based Health Promotion: Lessons from the Health Literacy Partnership

**DOI:** 10.3390/ijerph14121550

**Published:** 2017-12-11

**Authors:** Emee Vida Estacio, Mike Oliver, Beth Downing, Judy Kurth, Joanne Protheroe

**Affiliations:** 1 School of Psychology, Keele University, Staffordshire ST5 5BG, UK; 2 Stoke-on-Trent City Council, Public Health, Staffordshire ST4 1HH, UK; mike.oliver@stoke.gov.uk (M.O.); beth.downing@stoke.gov.uk (B.D.); 3 Centre for Health and Development (CHAD), Staffordshire University, Staffordshire ST4 2DF, UK; judy.kurth@staffs.ac.uk; 4 Research Institute for Primary Care & Health Sciences, Keele University, Staffordshire ST5 5BG, UK; j.protheroe@keele.ac.uk

**Keywords:** health literacy, effective partnerships, community-based health promotion, collaborative working

## Abstract

This paper aims to explore key elements needed to successfully develop healthy partnerships and collaborative working in community-based health promotion. It draws upon the lessons learned from a case study with the Health Literacy Partnership in Stoke-on-Trent, UK in developing the health literacy strategy in the area. The process was underpinned by respect for diverse yet complementary perspectives and skills from the grassroots up. This involved engagement with key stakeholders, development and support for community projects, and sharing of good practice with other national and local organizations. Stakeholders involved in developing the strategy also had a keen interest in health literacy and a strong commitment to promoting health and well-being in the area. Through patience, perseverance, and continuous open communication and learning, the health literacy strategy in Stoke-on-Trent, UK is beginning to have a ripple effect into local practice, and will potentially influence policy in the future.

## 1. Introduction

Partnerships between academics, public, and voluntary sector organizations are widely reported in the health promotion literature [1,2,3]. Although tensions and conflicts may arise due to the blurring of relationship boundaries [4,5], it is important to recognize that working in partnership with multiple agencies is crucial to the successful implementation and maintenance of community-based work [6,7].

Health as a concept is complex and the promotion of health requires consideration of its wider social, economic, cultural, and environmental determinants. Inter-agency partnerships can provide a holistic approach towards improving health and reducing inequities. Understanding the role of partnerships on how health promotion initiatives are designed, delivered, and maintained can also provide useful insights for researchers, practitioners, and policy makers when funding, planning, and evaluating such programs [8].

This paper draws upon the lessons learned from a case study with the Health Literacy Partnership in Stoke-on-Trent, UK in developing the health literacy strategy in the area. These lessons are transferrable to other contexts and can provide insight into effective and collaborative working in community-based health promotion. 

## 2. Context

Health literacy can be defined as the “personal characteristics and social resources needed for individuals and communities to access, understand, appraise, and use information and services to make decisions about health” [9]. The Health Literacy program described here was a Stoke-on-Trent City Council Public Health-led initiative which emerged from the UK Healthy Cities Network.

The health literacy partnership between academics at Keele University and Stoke-on-Trent City Council Public Health began in 2010 with a small exploratory project on health literacy and diabetes management [10]. This partnership grew and cascaded into more research projects, including an extensive (*n* = 1046) baseline survey of health literacy levels in the city and an assessment of the readability of health resources in the area. Findings suggest that 52% of the adult population in Stoke-on-Trent had less-than-adequate health literacy, and that it was associated with older age, poorer health, digital exclusion, and living in deprived areas [11]; whereas most patient information leaflets in General Practice (GP) surgeries were found to be too complex for 43% of the population [12].

These research findings have been influential in the development of the Stoke-on-Trent Health Literacy Strategy. They helped to define the problem and created a “sense of urgency” for change. They also helped to crystallize thinking from across a range of disciplines. Bringing different organizations together engendered a sense of hope that there is something that can be done to tackle the challenges of health literacy and that everyone has a role to play.

Research was used as a springboard for discussion in four large-scale annual community engagement events which raised the profile of health literacy in the city and generated ideas and commitment from local groups (see Table 1). The first workshop was designed specifically to create this sense of urgency and to rally support for creating change.

By engaging multiple stakeholders in the process, insights from research and practice grew. These were used to inform local policy and future action plans. Good project management in maintaining this engagement and some seed corn funding also enabled people to put their ideas into practice which gave sufficient “quick wins” to keep the partnership interested. Several activities and grassroots initiatives were developed and supported as a result (see Table 2).

Starting from a small partnership involving a core group with less than a dozen members, the Health Literacy Partnership now involves a whole host of individuals and organizations with a common agenda to improve health in Stoke-on-Trent by promoting health literacy in the city. Members of the partnership now include researchers, healthcare professionals (e.g., GPs, nurses, pharmacists, hospital managers, dentists), public health professionals, social workers, home care visitors, teachers, community education workers, volunteer organizations, housing agencies, community advocates, librarians, city planners, patient groups, and The Fire Service. Overall, the partnership involves a network of around 245 people who have been involved in training and community events in health literacy in Stoke-on-Trent.

This flourishing partnership was recognized for good practice in national reports, including the Inquiry Report into NHS England’s Five Year Forward View [13] and Public Health England’s report on local action on health inequalities [14], and was commended by the Phase VI (2014–2018) of the WHO European Healthy Cities Network [15].

Building upon and working alongside Health Literacy UK pioneers, plans were also made to collectively influence policy at a national level. Members of the Health Literacy Partnership in Stoke-on-Trent are also members of Health Literacy UK (one of whom is the Chair, and a Health Literacy Clinical Advisor to NHS England), and are also members of the NHS England Health Literacy Collaborative.

While there have been discussions in the literature around some of the challenges in community-based health promotion and collaborative working [16], this paper will contribute to this area of health promotion practice by highlighting key factors that contributed to the success of this collaboration.

## 3. Shared Passion for Reducing Inequalities in Health

The literature suggests that partnerships are most successful when there is a clear goal for the partnership [17] and that partners are working towards a shared vision [18]. In this case study, while the promotion of health literacy was the key driver, members of the partnership shared the wider agenda of reducing health inequalities in the area. While Stoke-on-Trent can be proud of its rich cultural heritage, health and social outcomes in the area can still be improved. The success of this partnership is rooted in the shared passion and commitment from those involved to promote the health and well-being of residents in the city.

Regardless of their specialist area and background, members of the partnership were passionate about reducing health and social inequalities and improving the community’s quality of life. The common agenda was to reduce health inequalities by improving patient experience, shared decision making, and self-management through health literacy. While improving the health literacy skills of patients was seen as important, members of this partnership also shared the same critical lens and recognized the importance of wider social and environmental factors that influence health.

From the original members of the steering group, to advocates joining in from the training and follow-up sessions, members of this partnership included a core group of committed enthusiasts who “get it” and “get on with it”. They are catalysts for change.

## 4. Diversity Requires Respect and Trust

The adage *“the whole is greater than the sum of its parts”* fittingly applies in this case. The Health Literacy Partnership in Stoke-on-Trent achieved more by working together than individual organizations could achieve on their own [18]. Although these organizations shared the same vision, the background and expertise brought forward by individual members were quite diverse. This diversity contributed to the partnership’s strength, since complementary knowledge, skills, and experiences were constantly being brought to the table.

For example, when collecting data to assess baseline health literacy levels in the city, academics in the team were able to advise on standardized measures that can be used for the project; city council partners were able to advise on potential barriers to recruitment; while grassroots leaders were able to advise on the best ways to engage community members.

Likewise, when developing and implementing health literacy programs, researchers were able to advise on robust methods to monitor and evaluate interventions, while service providers were able to advise on pragmatic ways to encourage uptake. Thus, maintaining a partnership that was equitable between community members and academics was vital in this process [19].

Strong public health leadership was also crucial in the early stages in engaging a wide range partners around a common set of community health promotion values and principles. This work built on the trust that had been established within the wide network of organizations that that the Healthy Communities team within the Public Health department has been working with as part of its remit. Equally important were the skills required to manage such a diverse network—particularly the project management skills from one of the core members of the partnership. Lack of awareness of the skill and capacity to manage this type of work often undermines it.

Involving multiple stakeholders from different backgrounds also required respect and trust to ensure that the partnership was sustainable and can achieve systemic transformations [20,21,22]. In this case, the growth and development of this collaboration was based on mutual trust from individual members and the understanding that the partners were contributing to the achievement of a common goal. There was also respect for the skills and expertise that members of the collaboration were contributing to the team.

Interactions in this partnership were often collegiate, and a great sense of respect and gratitude for the skills, expertise, and time offered by partners were often expressed. We believe that it is this spirit of co-operation that has led to the sustainability and on-going nature of this partnership. Strong partnerships are indeed created by our interactions with each other (i.e., our personal qualities) and the actions we take [5].

## 5. Learning, Networking, and Open Communication

Willingness to learn from one another is fundamental in establishing genuine partnerships such as this [21]. Considering the diversity of backgrounds in this partnership, it was inevitable that members would have different starting points in terms of knowledge and awareness of health literacy research, practice, and policy. Although some members were more familiar with the health literacy agenda than others, those who knew more were open to sharing, while those who knew less were open to learning.

Curiosity and eagerness to learn were matched by enthusiastic sharing of knowledge and ideas from research and practice. Meetings, events, and training opportunities were organized to cultivate learning and foster networking between members of the collaborative. Organizing these events also helped to encourage professional development and inter-disciplinary practice [23].

Clear and effective communication is another important feature of effective collaborations [24]. In this case, regular meetings were held, including an annual event to bring together the various organizations that were involved in this process. Regular email updates were also sent and workshops organized to foster further communication and learning.

## 6. Discussion

Partnership working in community-based health promotion can bring about fruitful and sustainable benefits for those involved. Although it takes some time to nurture relationships, when facilitated effectively, collaborative work can enable more systemic ways of working towards health promotion and community development.

As shown in the case study involving the Health Literacy Partnership in Stoke-on-Trent, committing to a shared vision, having mutual trust and respect for each other, and being open to share, learn, and communicate are vital elements that helped to make this partnership a success.

To maintain momentum, there is a need to sustain relationships and commitments. It is useful to continue to cultivate the knowledge and experience developed in this partnership so that other health promotion initiatives can also learn from this practice. The monitoring and evaluation of outcomes are particularly important, in order to ensure that efforts are recorded and recognized for their value to the community.

However, there are also threats to the sustainability of this partnership [25]. For example, availability of funding and the potential impact of staff and policy changes could be detrimental to the partnership’s future. We could be disrupted by policy reform, as well as by internal and external political influences (individual and organizational).

Thus, there is a need for organizational level commitment to uphold the health literacy agenda in Stoke-on-Trent. For a start, the Health Literacy Partnership in Stoke-on-Trent is driving forward the Health Literacy Friendly Project, which aims to develop a partnership approach to help organizations improve their entire system and environment when it comes to health literacy (e.g., communications, signage, layout of physical buildings, and policies). As [8] states, “if organizations from diverse sectors can embed a vision for health that accounts for place, complex health promotion initiatives may be less vulnerable to broader system reforms, and health in all policy approaches more readily sustained.”

Thus, embedding health literacy into the organizational culture in Stoke-on-Trent could buffer this threat by normalizing this as common practice, rather than it being something that only the health literacy aficionados do. One way to embed this into the organization might be to formalize this partnership as a specific work project with a specifically employed project manager. This would enable the practicalities of responsibilities for tasks; setting up of regular meetings and driving the agenda forward were taken on board. However, it is important not to neglect the diverse yet complimentary experience of the partnership, recognizing different strengths and different ways of working. One of the major benefits of this working partnership has been the space for both a shared vision, but also the flexibility that working together has led to, allowing accommodation of the visions and goals of individuals and different organizations involved.

## 7. Conclusions

As discussed in this paper, partnership working in community-based health promotion requires having a shared vision, mutual trust, respect, and openness to share and communicate. This involves engagement with key stakeholders, development and support for community projects, and sharing of good practice between organizations. As a result of the continuous support of the various members of this partnership, the health literacy strategy in Stoke-on-Trent is beginning to have a ripple effect into local practice that will potentially influence policy at local and national levels in the future.

## Figures and Tables

**Table 1:** Summary of key health literacy activities in Stoke-on-Trent.

Date	Location	Event	Purpose/Outcome
June 2014	Stoke-on-Trent Town Hall	Ideas Exchange	Raising awareness of the issue/challenge with local community groups and healthcare professionals. Capturing initial response and generating ideas
March 2015	The Bridge Centre, Stoke-on-Trent	From Ideas to Action	Building on the ideas and turning them into specific initiatives as part of a structured strategy
June 2016	Keele University	Update and Moving Forward	To hear progress on the projects and to stimulate debate on new ways of improving the health literacy environment in Stoke-on-Trent
June 2017	Hanley Library, Stoke-on-Trent	Is Stoke-on-Trent a health literacy friendly city?	To challenge ourselves—are we making progress? How can we empower people in addition to changing the health literacy environment?

**Table 2:** Summary of key health literacy initiatives in Stoke-on-Trent (2015–2017).

Organization	Initiative	Those Supported by This	Intended Outcomes
Stoke Speaks Out	Using Early Years Story Boxes, Stoke Speaks Out works directly with childminders, children’s centers, schools, nurseries, and parents to embed a range of early speech and communication strategies. Using play-based scenarios and a multi-sensory approach, the children are immersed in health-related vocabulary, which provides a foundation for later learning. So far, four health literacy Early Years Story Boxes have been created: (1) going to the dentist; (2) going to the hospital; (3) going to the doctors; (4) healthy eating	Early years children and their care providers	Increased familiarity and confidence with health scenarios and language
Open Network & Schools Sports Partnership	This partnership project within schools in the city adds value to the physical education and sport experience of primary-aged children in Stoke-on-Trent by embedding health literacy concepts into the way that School Sports Leaders (who are children themselves) encourage others to be more active and healthy	Children in primary and secondary schools	Easier to understand and act on information about healthy exercise and eating
Haywood Community Hospital	Based on health literacy best practice, the Centre is improving the well-being of people with arthritis and related conditions by (a) being thoughtful about how information is made available to people, (b) working in partnership with the local Public Health team to gain access to the latest health information, and (c) ensuring that volunteers working in the center receive training in health literacy	Inpatients and outpatients being supported by this hospital	Improved information sharing via the use of trained volunteers in the Patient Information Centre
University Hospital of North Midlands	University Hospital of North Midlands have launched the “It’s OK to Ask!” initiative, which encourages patients to engage more fully with health care professionals by asking three questions: “What is my main problem?” “What do I need to do?” “Why is it important I do this?”	Outpatients	Increased confidence to engage with medical staff via the “It’s OK to Ask!” initiative.
The Cultural Sisters	The Cultural Sisters is a participatory arts organization with a focus on Arts and Health, engaging with people using a creative processes to explore and learn about health and wellbeing issues. Health literacy concepts have been embedded into these arts projects, allowing people to engage more fully with the health messages being shared	Vulnerable groups in society	Increased esteem and confidence through participatory arts
Community Health and Learning Foundation	Health literacy training and awareness for a broad range of professions and service providers, including General Practitioners (GPs) and other GP practice personnel, pharmacists, dentists, school teachers and other educators, participatory art group leaders, social workers, local authority planners and commissioners, and fire service professionals. Training courses were also run alongside that equip other trainers and teachers to support service users, school children, and other people from Stoke-on-Trent with the confidence and knowledge to improve their own health literacy	GPs and other GP practice personnel, pharmacists, dentists, school teachers and other educators, participatory art group leaders, social workers, local authority planners and commissioners, and fire service professionals. 65 people trained, 3 courses delivered (Year 1); 92 people trained, 6 courses delivered (Year 2); 49 people trained, 3 courses delivered (Year 3)	Increased understanding of health and literacy and confidence in supporting service users to improve their own health literacy
Quality Improvement Framework	Quality Improvement Framework that includes health literacy as a key component and a health literacy video as part of the training.	GPs and other GP practice personnel, and the wider public	Increased understanding of health and literacy and confidence in supporting service users to improve their own health literacy
